# A Review: Grating Encoder Technologies for Multi-Degree-of-Freedom Spatial Measurement

**DOI:** 10.3390/s25196071

**Published:** 2025-10-02

**Authors:** Linbin Luo, Maqiang Zhao, Xinghui Li

**Affiliations:** Tsinghua Shenzhen International Graduate School, Tsinghua University, Shenzhen 518055, China; luolb24@mails.tsinghua.edu.cn (L.L.); zhaomq24@mails.tsinghua.edu.cn (M.Z.)

**Keywords:** multi-degree-of-freedom, grating encoders, high-precision measurement, system integration

## Abstract

In advanced manufacturing, nanotechnology, and aerospace fields, the demand for precision is increasing. Driven by this demand, multi-degree-of-freedom grating encoders have become particularly crucial in high-precision displacement and angle measurement. Over the years, these encoders have evolved from one-dimensional systems to complex multi-degree-of-freedom measurement solutions that can achieve real-time synchronization. There can also be high-resolution feedback. Its structure is relatively compact, the signal output is also very stable, and the integration degree is high. This gives it a significant advantage in complex measurement tasks. Recently, there have been new developments. The functions of grating encoders in terms of principle, system architecture, error modeling, and signal processing strategies have all been expanded. For instance, accuracy can be improved by integrating multiple reading-heads, while innovative strategies such as error decoupling and robustness enhancement have further advanced system performance. This article will focus on the development of two-dimensional, three-dimensional and multi-degree-of-freedom grating encoders, exploring how the measurement degrees of freedom have evolved, and emphasizing key developments in spatial decoupling, error compensation and system integration. At the same time, it will also discuss some challenges, such as error coupling, system stability and intelligent algorithms for integrating real-time error correction. The future of grating encoders holds great potential. Their applications in precision control, semiconductor calibration, calibration systems, and next-generation intelligent manufacturing technologies can bring promising progress to both industrial and scientific fields.

## 1. Introduction

Multi-degree-of-freedom (Multi-DOF) position measurement has become a fundamental enabler in advanced manufacturing and precision control systems [1,2,3,4,5,6,7] as key applications such as wafer-level lithography [8,9,10,11,12,13,14], nanoscale manufacturing [15,16,17,18,19], and aerospace assembly move toward submicron and nanometer resolutions [20,21,22,23,24]. The number of motion axes continues to grow [25], placing increasingly stringent demands on accuracy and stability [26,27,28,29]. This shift is driving a rapid evolution from traditional single-axis displacement sensing to simultaneous multi-DOF metrology [30,31,32,33], posing new challenges in optical layout [34,35,36,37,38,39], measurement dimensions [40,41,42,43,44], signal decoding [45,46,47], and structural integration. Laser interferometers, with their exceptional linearity and resolution [48,49,50], have long been widely used in the field of precision measurement [51,52,53]. However, their complex optical configuration [54,55,56,57], susceptibility to environmental interference [48,58,59,60,61], and limited integrability often hinder their expansion into full multi-DOF measurement tasks [62], particularly when both in-plane and out-of-plane sensing must be achieved or compact sensor integration is required [63,64,65]. Grating encoders provide a compact and stable alternative to laser interferometers [54,66,67], offering advantages such as high signal stability [68,69,70], wide bandwidth [45,71,72,73], and ease of integration [74,75,76]. By tracking the relative displacement between a moving grating and a reference grating, these systems generate interferometric signals [77,78,79], enabling real-time displacement and angle sensing [80,81,82]. They exhibit inherent robustness and adaptability [83,84,85], especially in confined spaces [30,86,87,88], distributed sensor configurations [89,90,91], and high-dynamic applications [79,92,93,94].

Compared with laser interferometers, grating encoders present several advantages that are particularly relevant for multi-DOF measurements [95,96,97]. Interferometers remain the benchmark for ultimate accuracy and traceability [98,99,100], but their reliance on long optical paths makes them highly sensitive to refractive index fluctuations [101,102], temperature gradients [103], and vibration [104], which requires strict environmental control [105,106,107]. Extending interferometric systems to multi-DOF often demands several beam paths and optical components [108,109]. This increases alignment difficulty [110], system size, and cost [111], while Abbe errors become significant when the measurement axis is displaced from the true motion axis [112,113]. These factors limit their practicality in compact or industrial settings. Grating encoders, by contrast, employ diffractive gratings as both scale and reference [114,115,116], allowing for compact optical layouts with shorter beam paths and reduced sensitivity to environmental disturbances. Their near-coincident detection of displacement and angular motions mitigates Abbe error [68,75,76,117,118], while their simplified mounting and alignment lower the barrier for system integration [68,71,119]. Although the ultimate accuracy of grating encoders is typically below that of state-of-the-art interferometers, their balance of resolution, robustness, and cost effectiveness makes them particularly well suited for multi-DOF applications in semiconductor lithography [120], precision machine tools [121], and large optical instruments [122]. However, these benefits necessitate stringent requirements on the grating’s manufacturing precision and structural integrity [123,124,125,126]. As the required measurement resolution approaches nanometer or even sub-nanometer levels [127,128,129], the impact of grating imperfections such as pitch non-uniformity [130,131,132], surface roughness [123,133,134,135], and alignment errors becomes increasingly significant [134,136,137,138,139]. Achieving high measurement accuracy and repeatability requires gratings with extremely high structural fidelity and dimensional uniformity [8,140,141,142]. As a result, advanced manufacturing processes are required [143,144,145,146,147,148], with laser interferometric lithography being a primary method for fabricating high-density diffraction gratings [149,150,151,152,153]. Therefore, advances in exposure uniformity [137,154,155,156], resist processing [157,158,159,160], and pattern transfer techniques are crucial to supporting the next generation of optical encoder technology for ultra-precision applications [161,162,163,164].

**Figure 1:**
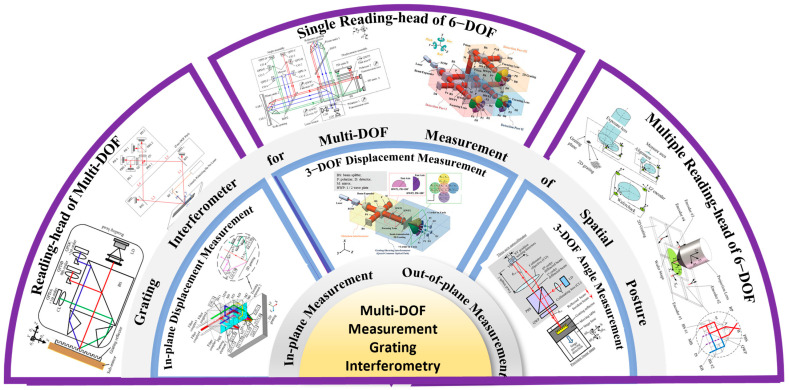
Classification and evolution of grating interferometry systems for multi-DOF spatial measurement [79,98,101,105,194,215,216,217,218].

As the required degrees of freedom for measurement continue to expand [86,165,166], optical grating encoders have evolved from early linear systems to increasingly complex two-dimensional [167,168,169], three-dimensional [96,170,171,172], and six-degree-of-freedom configurations [31,165,173,174,175]. Extensive research has explored a range of innovative technologies involving beam configurations [176,177], composite grating geometries [178,179,180], polarization modulation [181,182,183], digital signal processing [184,185,186,187], and error modeling [188,189,190]. Some systems achieve spatial decoupling by deploying multiple reading-heads [75,112,191], while others achieve high integration density by optimizing the internal optical path of a single sensor head [76,192,193,194]. To visually illustrate the evolution and classification of these systems, Figure 1 presents a conceptual diagram summarizing recent advances in multi-DOF grating interferometry [103,195,196,197,198,199,200]. We categorize the representative system architectures by dimension (in-plane and out-of-plane displacement, angle sensing), level of integration (single reading-head versus multi reading-head designs), and target degrees of freedom. This schematic framework reinforces the core logic of this review: a hierarchical comparative analysis along the three dimensions of measurement degrees of freedom, structural configuration, and system performance. To improve the readability of all diagrams in this paper, widely recognized components in precision metrology such as M (Mirror), BS (Beam Splitter), PBS (Polarizing Beam Splitter), PD (Photodetector), D (Detector), QPD (Quadrant Photodetector), NPBS (Non-Polarizing Beam Splitter), CL (Convex Lens) and QW (Quarter-Wave Plate) are labeled using standard abbreviations. For customized or application-specific elements, full terminology is retained to ensure clarity.

To accommodate the expanding variety of structural configurations and working conditions, traditional model-based decoding strategies are encountering growing limitations when dealing with nonlinearity [201,202], mechanical deformation [203], and environmental noise [19]. At the same time, the increasing availability of high-throughput sensing data from multi-channel encoders and high-speed detectors has enabled a shift toward data-driven signal processing [204,205]. In recent developments, neural networks and adaptive learning models have been introduced for signal estimation, error compensation, and pose reconstruction [206,207], offering enhanced robustness against structural misalignments and dynamic disturbances [208]. These learning-assisted techniques complement conventional optical and physical models and have shown promising performance in real-time multi-DOF sensing scenarios, particularly under complex or uncertain conditions. As a result, data-driven methodologies are emerging as a valuable enhancement to traditional encoder designs, contributing to higher accuracy and greater adaptability in precision metrology systems. Although previous reviews have discussed grating encoders from the perspectives of signal processing techniques and optical principles [80,209,210,211], they have seldom provided a systematic analysis of these systems in relation to the degrees of freedom they are designed to measure [212,213,214]. A focused reassessment that organizes the field around the evolution of measurable degrees of freedom is essential for clarifying technological trajectories, uncovering current limitations, and identifying opportunities for innovation. This paper therefore concentrates on recent advancements in grating encoders applied to two-dimensional, three-dimensional, and multi-axis displacement and angular measurements. Accordingly, this review is structured into four main sections. Section 2 examines grating encoder architectures for displacement measurement, covering normal-incidence and Littrow-incidence designs as well as absolute and multi-axis extensions, with critical comparisons of their resolution, range, and implementation complexity. Section 3 focuses on angular measurement using grating encoders, highlighting representative approaches based on diffraction, position-sensitive detection, and imaging strategies, together with their respective advantages and trade-offs. Section 4 discusses integrated multi-DOF spatial measurement encoders that combine translational and angular sensing within unified architectures, including single- and multi-reading-head designs, while also addressing coupling errors and error-compensation strategies. Finally, Section 5 summarizes the review with a forward-looking perspective, outlining key challenges and emerging opportunities, and highlighting future directions.

## 2. Displacement Measurement of Grating Encoders

### 2.1. Principle of Displacement Measurement

Grating-based interferometric systems are widely employed for high-resolution planar displacement measurement and are typically implemented using either homodyne or heterodyne configurations [219,220,221]. In heterodyne systems, a constant frequency offset is introduced between the two interfering beams [90,194,222]. The resulting beat frequency encodes the phase variations caused by displacement, allowing for unambiguous direction detection and enhanced immunity to noise [223]. However, this approach increases system complexity because it relies on active modulation and the presence of frequency demodulation circuitry. In contrast, homodyne systems obtain displacement information by measuring the phase difference between symmetrically diffracted beams, such as the first positive and negative diffraction orders, without introducing any frequency shift. Although homodyne configurations require more advanced phase unwrapping techniques to resolve directional ambiguity, they offer advantages in terms of optical simplicity, passive component usage, and ease of miniaturization. These characteristics make homodyne systems especially suitable for integration into compact multi-axis encoder heads where structural simplicity, operational stability, and high spatial resolution are essential.

**Figure 2:**
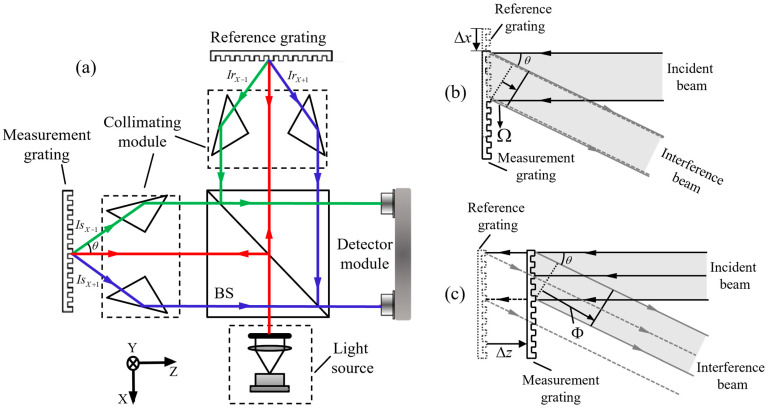
Fundamental principle of homodyne grating encoders for 2D displacement measurement [224]: (**a**) Schematic diagram of the overall optical measurement system; (**b**) Principle of in-plane displacement measurement using diffracted interference; (**c**) Principle of out-of-plane displacement measurement via optical path difference.

To illustrate the working principle of the principle of three-dimensional displacement measurement, a representative homodyne grating interferometer is considered. As shown in Figure 2, a collimated beam emitted by a laser diode is shaped into a parallel circular beam and directed through a beam splitter. The resulting beams are guided toward both a movable measurement grating and a fixed reference grating. When the measurement grating translates along either the X or Y axis, the first-order diffracted beams from both gratings experience phase shifts that correspond to the relative displacement between them. These beams are subsequently recombined and directed onto photodetectors, where interference signals are generated and recorded.

Two-dimensional displacement sensing is achieved by employing a grating with orthogonal periodic structures, which provides independent sensitivity along the X and Y directions. When the grating moves, the resulting optical path difference between the interfering beams leads to a phase shift that reflects the displacement along each axis. This shift, which can be described through analytical expressions, forms the basis for accurate measurement. By detecting and processing the interference signals through differential methods, the system achieves high-resolution sensing of planar motion at the nanometer scale.

As shown in Figure 2b, a collimated laser beam illuminates both the measurement and reference gratings. Each grating diffracts the beam into multiple orders, although only the first positive and negative orders are typically used to generate interference. These beams propagate symmetrically concerning the optical axis and overlap to produce an interference signal whose intensity varies with the relative displacement between the gratings. To facilitate planar measurement in two dimensions, the gratings are fabricated with periodic structures oriented along both the X and Y directions. When the measurement grating moves along either axis, the optical path difference between the interfering beams changes accordingly, resulting in a phase shift within the interference signal. For motion along the X direction, this phase shift between the first-order diffracted beams follows a linear relationship with the displacement and can be described analytically.

(1) $$\Omega_{X+1}=2\pi \frac{\Delta x}{g}$$

(2) $$\Omega_{X-1}=-2\pi \frac{\Delta x}{g}$$

where *g* is the grating period. An analogous relationship holds for the Y-direction displacement Δy:

(3) $$\Omega_{Y+1}=2\pi \frac{\Delta y}{g}$$

(4) $$\Omega_{Y-1}=-2\pi \frac{\Delta y}{g}$$

When the target grating moves along the Z-axis, the resulting displacement Δz induces a frequency shift in the diffracted beams. This frequency shift leads to a phase variation in the optical wave equation. As illustrated in Figure 2c, the induced phase $\Omega_{Z}$ is related to the grating displacement according to the following expression:

(5) $$\Phi =2\pi \frac{\Delta l}{\lambda }$$

(6) $$\Delta l=\Delta z\left(1+\cos\theta \right)$$

where *θ* denotes the diffraction angle of the grating.

The phase shifts induced by grating motion appear as intensity variations at the outputs of the photodetectors. The optical system typically includes two detectors, which collect the interference signals generated by the first positive and negative diffraction orders. In a full three-dimensional displacement scenario, the relative motion between the measurement and reference gratings along the X, Y, and Z axes contributes to changes in the optical path difference. These variations lead to corresponding phase shifts in the recombined beams. Assuming the gratings remain properly aligned and the diffracted beams have equal amplitudes, the interference intensities formed by the ±1st-order beams can be analytically described as functions of the three-dimensional displacement components. Both signals exhibit the same magnitude modulation. Directional information and quadrature signals are typically extracted using optical phase retarders or spatial encoding structures, and the resulting signals are further processed to reconstruct the full displacement vector.

(7) $$I_{X+1}=2U_{0}^{2}\left[1+\cos(\Omega_{X+1}+\Phi )\right]$$

(8) $$I_{X-1}=2U_{0}^{2}\left[1+\cos(\Omega_{X-1}+\Phi )\right]$$

(9) $$I_{Y+1}=2U_{0}^{2}\left[1+\cos(\Omega_{Y+1}+\Phi )\right]$$

(10) $$I_{Y-1}=2U_{0}^{2}\left[1+\cos(\Omega_{Y-1}+\Phi )\right]$$

where *U*_0_ is the amplitude of the reference light and the measurement light when interference occurs. Based on basic trigonometric identities, the displacement can be demodulated from the interference signals using the following expression:

(11) $$\Delta x=\frac{g}{4\pi }\left[\arccos\left(\frac{I_{X+1}-2U_{0}^{2}}{2U_{0}^{2}}\right)-\arccos\left(\frac{I_{X-1}-2U_{0}^{2}}{2U_{0}^{2}}\right)\right]$$

(12) $$\Delta y=\frac{g}{4\pi }\left[\arccos\left(\frac{I_{Y+1}-2U_{0}^{2}}{2U_{0}^{2}}\right)-\arccos\left(\frac{I_{Y-1}-2U_{0}^{2}}{2U_{0}^{2}}\right)\right]$$

(13) $$\Delta z=\frac{\lambda }{4\pi \left(1+\cos\theta \right)}\left[\arccos\left(\frac{I_{X+1}-2U_{0}^{2}}{2U_{0}^{2}}\right)+\arccos\left(\frac{I_{X-1}-2U_{0}^{2}}{2U_{0}^{2}}\right)\right]$$

In practical systems, the presence of a direct current (DC) component, which ideally corresponds to a stable background intensity, can have a significant impact on the accuracy of displacement demodulation. In reality, the DC level often fluctuates due to several factors, such as thermal drift in optical components and detectors, intensity imbalance between the interfering beams, and variations in diffraction efficiency along the optical path. These fluctuations can distort the normalized interference signal and introduce errors in phase demodulation, ultimately reducing the resolution of incremental displacement measurement. To mitigate these effects and enhance measurement stability, robust signal preprocessing techniques are typically employed. Common strategies include baseline compensation, high-pass filtering, and quadrature signal reconstruction.

Based on the fundamental principles of grating-based displacement sensing, the optical configuration plays a critical role in determining the system’s resolution, environmental tolerance, and integration potential. Among various implementations, two commonly adopted configurations in planar grating interferometry are the normal-incidence and the Littrow-incidence arrangements. These configurations differ in diffraction geometry, path symmetry, and susceptibility to external disturbances. A clear understanding of their operating principles and structural features provides a basis for comparing their performance and suitability in high-precision displacement measurement applications.

### 2.2. Representative Architectures of 2-DOF Displacement Grating Encoders

#### 2.2.1. Normal Incidence Grating Encoders for Displacement Measurement

**Figure 3:**
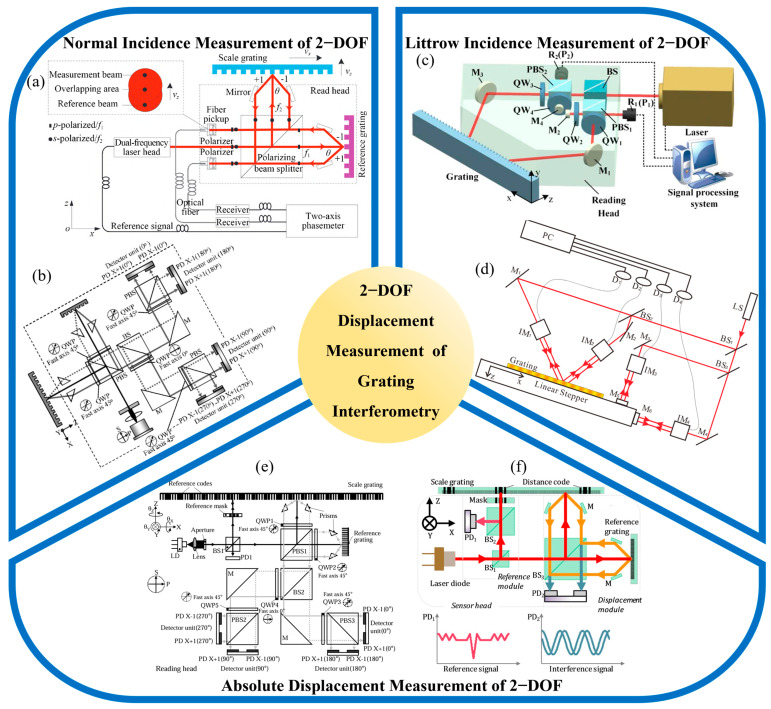
Representative structures of grating interferometers for 2-DOF planar displacement measurement with different incidence configurations: (**a**) Heterodyne two-degree-of-freedom system [228]; (**b**) Miniature differential dual-grating interferometric encoder [224]; (**c**) Symmetric Littrow-based in-plane encoder with Z-decoupling [215]; (**d**) Compact polarization-Littrow 2D encoder [229]; (**e**) Dual-probe hybrid positioning encoder [230]; (**f**) Window-fitting enhanced planar encoder [231].

In grating interferometry, the normal-incidence configuration refers to the optical arrangement in which a laser beam is directed perpendicularly onto the surface of a two-dimensional diffraction grating [225]. This setup typically generates first-order diffracted beams along orthogonal directions, which are used to form interference signals that encode planar displacements along the X and Y axes. Owing to its symmetric diffraction geometry and straightforward beam propagation, the normal-incidence configuration offers a compact and easily reconfigurable optical structure. It has been widely adopted in early encoder designs and planar motion systems. However, its non-common-path geometry makes it more vulnerable to phase noise caused by air turbulence, thermal drift, or out-of-plane disturbances along the Z-axis, all of which can affect long-term measurement stability. To address these challenges, practical implementations often incorporate techniques such as differential signal processing, multipath averaging, and environmental compensation. Representative structures of grating interferometers for 2-DOF planar displacement measurement are illustrated in Figure 3.

In 2007, Xia, H. developed a two-dimensional displacement measurement system based on moiré fringe analysis between planar gratings [226]. The system employed a normal-incidence configuration and achieved minimum measurement errors of 0.27 μm and 0.31 μm along the X and Y axes, respectively, within a 23 mm by 23 mm travel range. This work demonstrated the feasibility of applying grating-based interference to realize compact and practical two-dimensional encoders. In 2008, Hsu, C.-C. extended this concept by introducing a heterodyne grating interferometer capable of both one-dimensional and two-dimensional measurements using a single optical probe [227]. By combining active vibration isolation with frequency-shifted signal demodulation, the system achieved a displacement resolution of 0.5 nm over a 250 μm measurement range. This result highlighted the potential of achieving sub-nanometer sensitivity without sacrificing system compactness.

Building on these developments, as shown in Figure 3b, Gao, W. proposed in 2010 a differential grating interferometer that utilized first-order diffraction from both reference and scale gratings [224]. The optical head was miniaturized to 50 mm by 50 mm by 30 mm while maintaining sub-nanometer resolution, confirming the viability of compact design without compromising accuracy. Further expanding the flexibility of grating interferometry, as shown in Figure 3a, in 2014, Zhu, Y. proposed a heterodyne interferometric system designed to simultaneously measure long-range in-plane and short-range out-of-plane displacements [228]. The system achieved resolutions of 1.63 nm in the X direction and 0.75 nm in the Z direction, with standard deviations of 6.37 nm and 3.69 nm under repeated tests. This work demonstrated the adaptability of grating-based systems for multi-dimensional and multi-scale precision measurement tasks.

In 2015, Lin, C. focused on improving diffraction efficiency and signal contrast in normal-incidence grating structures. Through optimization of polarization alignment and grating parameters, the system achieved a diffraction efficiency of 13.82% and a signal contrast ratio of 100% [232]. These improvements significantly enhanced resistance to angular misalignment and signal degradation, making the system more suitable for high-dynamic and multi-axis measurement environments. In 2019, Zhang, M. introduced a compact two-dimensional grating encoder that integrated heterodyne demodulation within an optimized optical layout [233]. This design eliminated the need for mechanical rotation and enabled efficient signal extraction. The experimental results showed minimum resolvable displacements of 0.26 nm along the X axis and 0.465 nm along the Z axis, confirming its capability for high-resolution displacement feedback in confined installations. Most recently, in 2022, Li, W. developed a dual-path normal-incidence grating interferometric system that employed spatially separated optical channels for the reference and measurement beams [79]. This configuration improved phase stability and signal isolation. The system achieved a resolution better than 3 nm, with displacement errors confined within ±50 nm (X-axis) and ±100 nm (Y-axis) over a 40 mm range, meeting the requirements of long-range, high-precision applications.

These systems are now increasingly integrated into semiconductor manufacturing, photonics assembly, and precision metrology platforms. Despite their advantages, however, normal-incidence configurations still face inherent limitations. Although out-of-plane displacement can be partially addressed through structural calibration or mathematical compensation, the effective measurement range along the Z axis remains constrained by the diffraction geometry and optical symmetry. This restricts their direct use in applications involving large out-of-plane motion or significant angular variation. In addition, the overlapping optical paths of reference and measurement beams increase sensitivity to alignment errors, which can compromise robustness in multi-DOF environments.

#### 2.2.2. Littrow Incidence Grating Encoders for Displacement Measurement

Compared with normal-incidence configurations, Littrow-incidence grating interferometers offer improved robustness against out-of-plane displacement due to their folded optical geometry [75,234]. In this arrangement, the incident and diffracted beams propagate in opposite directions along the same path, forming a retro-reflected structure. This design not only enhances measurement sensitivity through a double-pass effect but also reduces coupling to Z-axis motion. The configuration further facilitates spatial separation of detection channels and supports compact packaging, which is advantageous for high-precision 2D and 3D displacement sensing.

As shown in Figure 3d, In 2016, Lu, Y. proposed a compact two-axis optical encoder based on a symmetric Littrow configuration and a high-density grating with a 561.8 nm pitch [229]. The system integrates two heterodyne interferometric modules to extract horizontal and vertical displacements simultaneously. By calibrating the average angle between the grating vector and motion direction, cosine errors were mitigated. The experimental results showed strong agreement with a commercial laser interferometer, with maximum deviations of 60 nm (horizontal) and 250 nm (vertical) over an 8 mm travel range, and sub-nanometer differences within 20 nm-scale displacements. Static tests confirmed sub-nanometer noise over 3 s, and a resolution of 0.137 nm was achieved via combined optical and electronic subdivision. This design demonstrates high precision and stability suitable for integrated 2D displacement sensing. As shown in Figure 3c, in 2018, Lv, Q. presented a symmetric Littrow-incidence encoder that employed a single reflective grating illuminated by two incident beams at equal and opposite angles [215]. The ±1st-order diffracted beams interfered at the detector, and the resulting phase shifts were used to reconstruct in-plane displacement. The system achieved a resolution better than 1 nm, with repeatability below 10 nm. To verify its resistance to out-of-plane drift, the authors introduced a vertical displacement of 1 mm, which resulted in an X-axis position error of less than 0.08 nm. This confirmed the intrinsic decoupling capability of the Littrow configuration concerning Z-direction perturbations.

In 2021, Yin, Y. further advanced the concept by implementing a dual-beam Littrow encoder with polarization-resolved detection and digital phase demodulation [193]. Two linearly polarized beams were introduced at opposite Littrow angles, and the return beams were separated using a Wollaston prism for independent detection. The system incorporated an 8-level digital phase interpolation algorithm to improve resolution and suppress nonlinearity. It achieved a resolution of 0.3 nm, with linearity error kept below 0.5% across ±1 mm measurement range. Repeatability tests showed standard deviations within 2.5 nm along both axes, demonstrating consistent performance under laboratory conditions.

Collectively, these studies confirm the effectiveness of Littrow-incidence configurations in achieving sub-nanometer in-plane resolution while suppressing sensitivity to out-of-plane displacement. Their use of angularly selective beam paths, polarization multiplexing, and high-precision demodulation supports compact designs without compromising performance. To further illustrate the structural differences and trade-offs between Littrow and normal-incidence geometries, a comparative summary is provided in Table 1. As the table shows, while the Littrow configuration offers superior robustness to Z-direction perturbations and greater angular tolerance, it also presents practical limitations. Precise alignment of incidence angles is essential to maintain optical symmetry and diffraction efficiency. Reflective gratings require tight fabrication tolerances, and although the folded geometry supports compact integration, it can limit layout flexibility in multi-sensor assemblies. Overcoming these constraints is key to expanding the application of Littrow-based encoders in advanced industrial settings.

A comparison between normal-incidence and Littrow-incidence grating encoders highlights the inherent trade-offs that must be considered when selecting an optical architecture. Normal-incidence systems are structurally simple and relatively easy to align, which makes them attractive for compact and cost-sensitive platforms. They are capable of nanometric or even sub-nanometric resolution, but their non-common-path geometry and overlapping optical channels render them sensitive to thermal drift, turbulence, and misalignment. These factors limit long-term stability and restrict the effective range, particularly along the Z axis. Littrow-incidence encoders, by contrast, suppress sensitivity to out-of-plane disturbances through folded optical paths and double-pass beam geometry. They achieve sub-nanometer resolution with good angular tolerance, and their retro-reflective layout facilitates multi-DOF extension. However, these benefits come at the cost of increased design and fabrication complexity. Precise angular alignment is required to preserve symmetry, and reflective gratings demand high surface quality and strict manufacturing tolerances. While the folded geometry allows for compact packaging, it also reduces flexibility for integration in multi-sensor assemblies.

From a practical perspective, normal-incidence encoders are better suited to scenarios where simplicity, low cost, and moderate resolution are the primary requirements, such as compact planar motion stages or photonics assembly tools. Littrow-based designs are more appropriate in applications that demand high resolution and robustness to environmental perturbations, such as semiconductor lithography and ultra-precision machining, even though they require greater implementation effort. This contrast illustrates that no single configuration offers optimal performance in all aspects. Instead, resolution, measurement range, system complexity, and implement ability must be balanced according to the demands of the target application.

#### 2.2.3. Absolute Grating Encoders for Displacement Measurement

While the periodic nature of phase signals causes most grating interferometers to operate in incremental mode, the growing demand for real-time absolute positioning in fields such as semiconductor manufacturing and precision motion control has driven the development of absolute planar grating encoders [235]. These systems combine grating-based interferometry with advanced encoding strategies to determine the unambiguous position of a moving grating in two dimensions [236].

Early approaches typically relied on multi-track encoding schemes. In 2003, Matsuzoe, Y. proposed a method that combined M-code with multi-period signals to derive absolute position by interpolating phase data from two grating tracks with different pitches [237]. Although effective in achieving absolute decoding, this method introduced challenges related to track fabrication and alignment, and often required wider grating areas. To simplify the structure, later research focused on single-track quasi-absolute encoding, where absolute marks are embedded directly into the incremental grating. These marks, usually implemented as reference pulses with unique width or spacing, provide coarse position information that is further refined through high-resolution phase interpolation. In such designs, the construction of the absolute code plays a critical role, as it must enable reliable decoding within limited spatial dimensions while maintaining the integrity of the primary interference signal.

In 2016, Li, X. developed a dual-probe optical encoder based on a single-track grating embedded with multiple reference marks of varying widths and duty cycles [238]. These marks were directly integrated into the incremental grating grooves without requiring additional tracks, allowing for a compact design. The system achieved an absolute positioning accuracy of 0.5 μm, with a linearity error below 0.06%, demonstrating the feasibility of quasi-absolute measurement with high repeatability and low structural complexity. Building on this concept, as shown in Figure 3e, Shi, Y. in 2019 introduced a hybrid-positioning method using two optical probes [230]. One reading-head, combined with a coded mask, identified coarse position from reference patterns, while the second head measured displacement with nanometric resolution via standard grating interferometry. Experiments showed a repeatability of 10 nm over travel distances of several tens of millimeters, confirming effective decoupling between the absolute and incremental channels. As shown in Figure 3f, further refinements in 2020 included a window-fitting algorithm for precise peak detection of reference pulses, pushing the incremental resolution to 15 nm, with absolute accuracy approaching this level [231]. These developments reflect a growing interest in compact absolute planar encoders that offer high integration density and minimal signal interference.

To meet the increasing demands on resolution and robustness, researchers have explored computational and learning-based methods for encoding optimization. Traditional exhaustive search approaches become impractical as the number of bits increases. In 2023, Wang, S. proposed a code-coupling optimization framework that jointly tuned key system parameters, including the mask-to-grating distance, incident angle, and unit width [239]. These parameters were integrated into a multi-variable model to simulate and evaluate encoding performance. The resulting code structures provided micron or sub-micron accuracy while offering flexibility for system-level integration. In the same year, the team introduced a genetic algorithm-based binary code design method, which automatically generated high-performance encoding patterns suitable for nanometric resolution. This approach significantly improved both positioning accuracy and design efficiency compared to manual coding methods. In 2024, they further advanced this line of research by employing generative adversarial networks to generate optimized 150-bit binary zero-position encodings [240]. Compared with conventional binary designs, the proposed method improved decoding accuracy by more than 129% relative to conventional binary designs, offering a scalable and intelligent solution for future absolute encoder development.

Collectively, these efforts indicate that absolute planar measurement is no longer restricted to traditional full-track gray codes or M-code patterns. Instead, the integration of hybrid optical architectures with algorithmically optimized encoding schemes enables compact, high-resolution 2D absolute encoders. These systems combine the repeatability and nanometric precision of grating interferometry with reliable coarse-position anchoring, making them highly suitable for precision metrology and advanced motion control applications.

### 2.3. Representative Architectures of 3-DOF Displacement Grating Encoders

**Figure 4:**
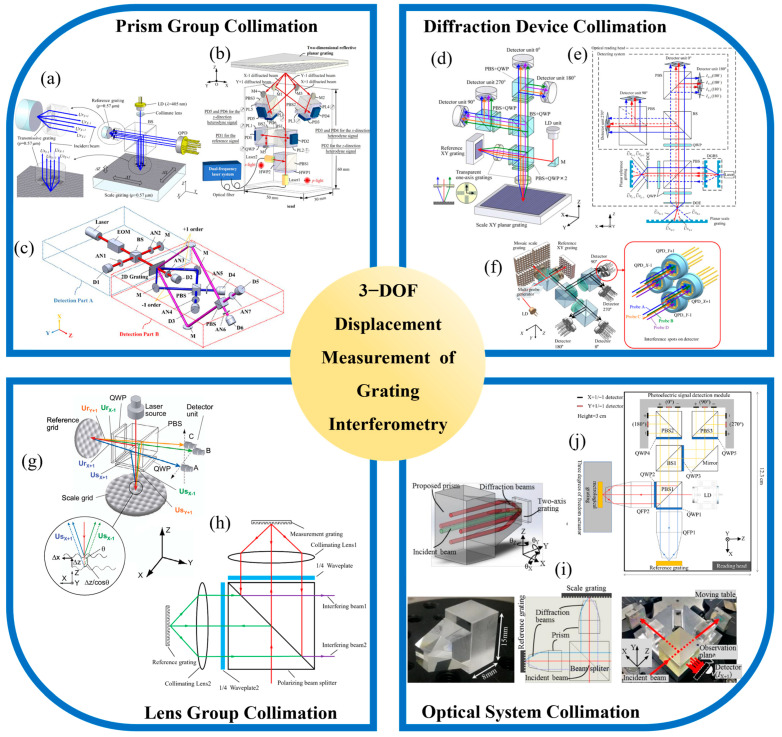
Representative collimation architectures for 3-DOF measurement grating encoder: (**a**) Compact 3-DOF grating interferometer [241]; (**b**) Normal-incidence heterodyne 3-DOF grating interferometer [217]; (**c**) Quasi-common-path symmetric heterodyne interferometer [170]; (**d**) Three-axis encoder with integrated reference grating [188]; (**e**) Mosaic-grating large-range grating encoder [97]; (**f**) Diffractive optical compact grating encoder with extended Z range [221]; (**g**) Sinusoidal-grating 3-DOF displacement encoder [242]; (**h**) Long-working-distance multi-DOF grating encoder [243]; (**i**) Quadrangular beam-parallelizing prism for 3-DOF grating interferometry [244]; (**j**) compact 3-DOF grating encoder with QFP-based beam parallelization [245].

Building upon the planar displacement measurement discussed in the previous section, three-degree-of-freedom grating interferometry extends the sensing capability of the system to include out-of-plane motion along the Z axis. This advancement is critical for applications such as wafer alignment, ultra-precision machining, and nano positioning, where full spatial motion tracking is required. However, incorporating Z-direction sensing into in-plane measurement systems introduces additional complexity, particularly in optical path design, signal decoupling, and crosstalk suppression. To address these challenges, researchers have investigated a range of collimation architectures capable of enabling simultaneous multi-axis interference while preserving both resolution and system stability. As shown in Figure 4, current implementations of three-degree-of-freedom grating interferometry typically adopt one of several representative collimation strategies. These include prism-based configurations, diffraction-based structures, lens-assisted systems, and compact integrated optical modules. Each approach involves distinct trade-offs in terms of structural compactness, beam overlap control, and alignment flexibility.

#### 2.3.1. Prism Group Collimated Grating Encoders

Prism-based collimation structures are among the most widely adopted configurations in three-degree-of-freedom grating interferometry, owing to their stable beam alignment, modular design, and strong resistance to environmental disturbances. These systems typically employ a combination of beam-splitting and retro-reflecting prisms to generate orthogonal diffraction paths, enabling simultaneous displacement measurements along the X, Y, and Z axes with high precision.

As shown in Figure 4a, in 2012, Li, X. introduced a compact 3-DOF grating interferometer using a pentaprism and a corner cube assembly to direct diffracted beams into separate interference channels [241]. The system achieved sub-micrometer resolution with nanometric repeatability in all three axes, laying the foundation for multi-axis grating-based metrology systems. Later, as shown in Figure 4b, in 2013, A grating-based heterodyne interferometer was developed for 3-DOF displacement measurement [217], combining the advantages of heterodyne and grating interferometry. A heterodyne beam generated via electro-optic modulation is normally incident on a 2D transmission grating. The resulting optical configuration enables simultaneous detection of in-plane (X, Y) and out-of-plane (Z) displacements. The system achieves nanometer-level resolution and millimeter-scale range without requiring changes in setup. The experimental results confirm its capability for precise multi-axis displacement and straightness measurement, with stable performance and effective suppression of alignment-related errors.

As shown in Figure 4c, in a separate study from 2022, Zhu, J. developed a quasi-common-path heterodyne grating interferometer using a symmetric reflective layout. The optical design shared multiple components across two beam paths, reducing sensitivity to thermal drift and mechanical instability [170]. The system employed a grating-based interferometric layout in which incident beams were split and recombined through a symmetric prism configuration. Digital phase-locked loop algorithms were used for real-time phase extraction, while fringe linearization and offset correction techniques enhanced signal fidelity. The system achieved resolutions of 0.18 nm along X and Y and 0.32 nm along Z, over a motion range of ±5 mm. It demonstrated strong robustness against vibration and alignment errors, supporting sub-nanometer repeatability in advanced manufacturing contexts. To further address nonlinear phase errors inherent in dual-frequency heterodyne systems, the same research group later proposed a wavelength-stabilized, quasi-common-path interferometer [246]. This design employed two spatially separated laser beams with a small frequency offset, stabilized to a rubidium atomic transition, and directed through a symmetric oblique-incidence prism configuration. This approach reduced periodic nonlinear errors to below 0.3 nm, while improving overall resolution, repeatability, and resistance to environmental fluctuations. The system proved well suited for ultra-precision positioning applications.

Prism-based collimation remains the most established solution for 3-DOF grating interferometry, benefiting from mature optical designs, strong alignment stability, and proven capability to enable simultaneous high-resolution measurements along three spatial axes. By combining corner cubes, beam splitters, and polarization optics, these systems achieve robust separation and recombination of multiple diffraction paths. Further enhancements including wavelength stabilization, quasi-common-path design, and error-compensation algorithms have continued to improve system precision and robustness. Nevertheless, prism-based architectures also present certain intrinsic limitations. Their optical layouts are often bulky and complex, requiring meticulous alignment to maintain measurement orthogonality. In particular, the measurable range along the Z axis may be constrained by angular sensitivity and diffraction geometry, which limits their suitability for applications involving large out-of-plane travel. Additionally, non-common optical paths, though partially compensated, may still introduce residual phase noise when exposed to environmental disturbances.

#### 2.3.2. Diffraction Device Collimated Grating Encoders

Diffraction-based collimation structures offer a compact and robust alternative to prism-aligned interferometric systems, particularly well suited for three-degree-of-freedom displacement measurements. These configurations utilize the angular selectivity of diffraction gratings to establish fixed, self-collimating optical paths that enable precise sensing while minimizing sensitivity to out-of-plane motion along the Z axis. Some systems employ Littrow-type diffraction geometries, which further reduce optical path length and support quasi-common-path operation, thereby improving thermal and mechanical stability.

As shown in Figure 4d, one of the earliest representative systems was introduced by Kimura, A. in 2012, who developed a three-axis surface encoder capable of sub-nanometer resolution [188]. The encoder used a planar XY grating composed of orthogonal 1 μm period structures fabricated through two-beam interference lithography, along with a matching reference grating integrated within the sensor head. Displacement along the X and Y axes was measured based on interference between the +1st-order diffracted beams from the two gratings, while Z-axis motion was derived from the optical path difference of reflected beams, using the laser wavelength as the effective scale. Experimental validation demonstrated resolution better than 1 nm in all three axes, with interpolation errors within ±10 nm (X/Y) and ±3 nm (Z). The system maintained a Z-directional working distance tolerance of ±150 μm, and angular misalignment tolerances of ±100 arcsec (X/Y) and ±200 arcsec (Z), indicating strong mechanical robustness. Expanding on the principle of diffraction-based alignment, as shown in Figure 4f, in 2014, Shimizu, Y. proposed a new configuration that incorporated a mosaic planar grating and a four-probe sensor head to increase the in-plane measurement range [221]. The mosaic grating was composed of multiple reflective grating segments arranged in a matrix, enabling seamless stitching of measurements over extended XY travel. The optical head employed four probes generated through a geometric layout involving beam splitters and laser diodes, each capable of simultaneously detecting displacement in the X, Y, and Z directions. Experimental evaluation confirmed sub-nanometer resolution across all axes, with nonlinearity errors within 5 nm at a 95% confidence level. Furthermore, the successful integration of multiple segments validated the system’s potential for large-range high-precision metrology.

More recently, as shown in Figure 4e, in 2017, Lin, J. et al. introduced a compact three-axis encoder that integrated an 8 μm pitch planar reflective grating [97], a reference grating, and advanced optical components including a double-grating beam-splitting unit and diffractive optical elements. This system utilized retro-reflected +1st-order beams as auto-collimating signal carriers, significantly extending the Z-axis measurement range while maintaining a compact overall footprint. By applying scalar diffraction theory to optimize beam shaping and optical efficiency, and implementing a custom 80-segment electronic division circuit, the system achieved a Z-axis resolution of 4 nm. A comprehensive analysis of alignment tolerance and nonlinearity was also performed, underscoring the system’s potential for integration into high-precision motion platforms. In recent years, additional research has contributed to the advancement of diffraction-based optical components. Developments in compact diffractive beam splitters and high-efficiency planar gratings have further improved system miniaturization and long-term stability, supporting broader deployment in space-limited and thermally sensitive environments.

In summary, diffraction device collimated grating encoders provide a structurally compact and optically stable solution for three-axis displacement measurement. The self-collimating behavior of diffracted beams renders the system highly insensitive to Z-axis misalignment, reducing dependence on bulky optics and facilitating easier system integration. Furthermore, the use of planar reference gratings simplifies fabrication and supports long-term thermal and mechanical reliability. Despite these strengths, diffraction-based systems face limitations in terms of sensitivity to grating surface imperfections, reduced Z-axis range relative to prism-based alternatives, and challenges in maintaining high interference quality over large areas. Nevertheless, ongoing improvements in diffraction component design and system-level integration continue to strengthen the case for these systems in high-resolution, multi-axis metrology for precision engineering applications.

#### 2.3.3. Lens Group Collimated Grating Encoders

Lens group collimated grating encoders use the focusing and collimating properties of a lens to align the diffracted beam, enabling configurations with longer working distances and larger fields of view. Unlike prism- or diffraction-based systems, these designs can accommodate longer optical paths by placing the measuring grating near the focal plane of the convex lens. However, a major limitation of this architecture lies in its pronounced sensitivity to axial displacement; even slight deviations along the Z axis can lead to noticeable defocusing and degraded signal quality. As such, it is better suited for applications that allow for more flexible optical layouts or require greater mechanical clearance, provided that sufficient control over Z-axis positioning is ensured to maintain measurement stability.

As shown in Figure 4g, in 2007, Gao proposed a three-axis displacement sensor that employed sinusoidal XY-grid mirrors in a lens-collimated configuration [242]. Instead of using conventional plane mirrors, the setup incorporated two-dimensional sinusoidal gratings with a 10 μm pitch and 60 nm amplitude as both reference and scale gratings. By analyzing the interference between their ±1st-order diffracted beams, simultaneous displacement measurements along the X, Y, and Z axes were achieved with nanometric resolution. The system demonstrated a Z-axis resolution of approximately 4 nm, with measured nonlinearity errors of 100 nm (X), 150 nm (Y), and 4 nm (Z). The optical system included a laser diode, polarizing beam splitters, and quarter-wave plates to generate quadrature signals, and enabled 5 nm step detection along both the X and Z axes.

To overcome limitations in working distance and prism alignment complexity, Luo et al. introduced in 2024 a multi-DOF grating encoder based on large-period gratings and long-focus plano-convex lenses [247]. The encoder achieved a working distance exceeding 80 mm and supported multi-DOF measurement by combining interference-based displacement sensing with autocollimation-based angular detection. Four diffracted beams from a two-dimensional grating were used to generate interference signals for in-plane motion tracking, while angular displacement was derived from the shifts in the diffraction spots. The system offered a simple structure, ease of installation, and tunable working distance by adjusting the focal length of the lens. Simulation results confirmed its feasibility, indicating its potential for integration into large-scale precision systems. As shown in Figure 4h, in the same year, the same group proposed another multi-DOF grating encoder based on lens-guided diffraction and quadrant photodetection [243]. The system was capable of high-precision simultaneous measurement of XY displacements and angular tilts around the X and Y axes. In-plane displacements were obtained using standard grating interferometry, while angular motion was detected by tracking the drift of diffraction spots on a QPD. The experimental results showed an error of ±2 μm within an 8 mm travel range, demonstrating high precision and repeatability. The authors emphasized the system’s potential for motion feedback and compensation in precision manufacturing, and noted opportunities for future improvements in modeling and dynamic performance.

Lens group collimated grating encoders offer a promising approach for long-range and multi-DOF measurement, particularly when large working distances or compact structural integration are required. Their advantages include structural simplicity, flexible optical alignment, and tunability based on lens parameters. However, their inherent sensitivity to Z-axis motion due to focal constraints limits their robustness in environments affected by axial drift or vibration. Ongoing research has focused on optimizing lens-grating configurations, incorporating angular tracking capabilities, and enhancing error resilience. These developments continue to position lens-based designs as a valuable complement to prism and diffraction-based architectures in precision metrology.

#### 2.3.4. Optical System Collimated Grating Encoders

Grating encoders based on optical system collimation employ customized optical modules to parallelize diffracted beams from multi-axis gratings. This approach integrates beam deflection, collimation, and alignment compensation into a compact architecture, enabling high integration and reduced mechanical complexity. Compared with traditional lens- or prism-based collimation methods, these systems offer distinct advantages in terms of size reduction, modularity, and mechanical stability. However, they typically depend on precise optical geometries and require the grating to be positioned near a specific focal plane, which limits their tolerance to out-of-plane displacement along the Z axis. In addition, the use of custom-fabricated optics increases both system cost and integration difficulty.

As shown in Figure 4i, in 2018, Li, X. proposed a novel collimation component for grating interferometry known as the quadrangular frustum pyramid prism [244]. Designed to parallelize four diffracted beams from a two-axis grating structure, the prism guides the incident beams through symmetric internal reflections and outputs them in parallel through its bottom surface. A prototype prism, capable of handling a 1 mm beam diameter and achieving 10 mm output beam spacing, was fabricated and tested. Experimental validation within a grating interferometer confirmed the device’s ability to maintain beam parallelism, reduce optical power loss, and simplify the assembly process. This advancement significantly improved the feasibility of integrating compact grating encoders. Building on this concept, as shown in Figure 4j, Wang, S. developed in 2023 a compact 3-DOF grating encoder incorporating the QFP prism design [245]. The system featured a miniaturized sensor head measuring 12.3 × 7.7 × 3 cm^3^, capable of simultaneously measuring displacements along the X, Y, and Z axes. The working ranges were ±250 μm in X, ±200 μm in Y, and ±100 μm in Z, constrained by the physical size of the grating. Measurement accuracy was reported to be better than 500 nm on average, with relative errors ranging from 0.0708% to 2.842%. The study also proposed further improvements, including enhanced grating fabrication and signal stabilization techniques, to extend the measurement range to the millimeter-scale and achieve accuracy within 0.1%.

Overall, the selection of a collimation architecture should consider a comprehensive balance between integration level, tolerance to alignment errors, and system design complexity. Each architecture presents distinct advantages and trade-offs that must be evaluated in the context of specific application requirements. To provide a clearer comparison, Table 2 summarizes the representative characteristics of different collimation architectures, including their structural configurations, alignment tolerances, and typical application scenarios.

The four collimation strategies for 3-DOF grating encoders illustrate distinct balances between resolution, measurement range, design complexity, and practical implement ability. Prism-based configurations provide mature and stable optical layouts with sub-nanometer resolution, but their bulky assemblies and limited Z-axis working distance restrict use in space-limited or long-range applications. Diffraction-based collimation achieves compact integration and reduced sensitivity to Z-axis drift, making it attractive for high-precision motion stages, yet its performance depends strongly on grating quality and the available Z-axis range remains more limited than in prism systems. Lens-group architectures allow for long working distances exceeding tens of millimeters and support additional angular measurements, but their pronounced sensitivity to axial displacement imposes strict control requirements that complicate deployment in vibration-prone environments. Optical system collimation offers high integration and modularity, enabling compact encoder heads suitable for system embedding, though reliance on custom-fabricated optics raises cost and reduces tolerance to assembly errors.

From an engineering standpoint, these trade-offs imply that no single configuration is universally optimal. Prism-based systems are preferred where ultimate resolution and stability are paramount, diffraction-based encoders are suited for compact and thermally stable instruments, lens-based approaches address scenarios requiring large clearances or extended ranges, and optical-system collimation supports highly integrated platforms. The practical adoption of each design therefore depends on prioritizing resolution, range, or compactness according to the demands of the target application, and future progress will likely involve hybrid or reconfigurable schemes that combine these advantages while mitigating current limitations. This overview helps guide the selection of an appropriate collimation strategy based on practical performance demands. In addition, emerging approaches such as hybrid collimation schemes or reconfigurable optical modules are being explored to overcome existing limitations, aiming to support the development of next-generation compact and high-performance grating encoder systems.

### 2.4. Artificial Intelligence Techniques for Enhanced Signal Processing in Grating Encoders

To meet the growing demands for precision and robustness in multi-DOF grating encoders, recent research has explored advanced data-driven techniques for signal decoding and error compensation [248]. These methods offer enhanced adaptability and computational intelligence compared to traditional static correction approaches, especially under conditions involving mechanical deformation, signal distortion, and dynamic measurement environments.

In 2019, Zhu, W. introduced a sinusoidal error compensation method based on particle swarm optimization (PSO), implemented on an FPGA platform to address signal distortion in moiré fringes [249]. They developed a harmonic waveform fitting model and used PSO to solve the waveform parameters efficiently, reducing the subdivision error from 0.95 arcsec to 0.56 arcsec, thus significantly enhancing angular resolution and reducing spectral noise in real-time applications. Then, in 2021, Li, Y. focused on displacement estimation challenges arising from the flexible deformation of lightweight motion stages [250]. To estimate the displacement of the point of interest (POI) without assuming rigid-body dynamics, a dynamic neural network (DNN)-based soft sensor model was proposed. Among multiple network topologies evaluated via a stepwise-weighted analysis, the DNN with hidden feedback achieved a correlation coefficient above 0.998 and relative errors within 5%, outperforming traditional least-squares models in both accuracy and adaptability.

In 2024, Li, R. addressed signal integrity degradation due to limited grating line pairs in compact reflective encoders [251]. They developed a real-time correction method targeting DC offset, amplitude imbalance, and quadrature phase error in moiré signals. Using an improved CORDIC-based arctangent interpolation, the subdivision error was reduced from 45 arcsec to 6 arcsec, achieving a measurement accuracy of ±1 μm, superior to commercial encoder performance under the same grating configuration. In 2025, Wu, Z. explored encoding error compensation in variable-speed measurement environments [252], which introduce complex speed-dependent signal distortions. They proposed a deep Q-learning reinforcement learning framework, mapping the 2D image-based compensation task to a 1D action-reward model. The system adaptively eliminated speed-related encoding errors and improved localization and decoding accuracy across multiple test scenarios, especially at low speeds from 0.10 mm/s to 0.50 mm/s.

Recent studies have demonstrated the potential of artificial intelligence in improving the signal quality and error compensation performance of grating encoder systems. Techniques such as dynamic neural networks, particle swarm optimization, and deep reinforcement learning have been successfully applied to address issues like signal distortion, structural deformation, grating tilt, and speed-related decoding errors. These methods contribute to better accuracy, adaptability, and robustness under varying conditions. Future efforts should focus on real-time implementation, improved generalization across hardware configurations, and enhanced resistance to external disturbances, paving the way for more intelligent and reliable encoder-based metrology systems.

## 3. Multi-DOF Angle Measurement of Grating Encoders

### 3.1. Basic Principle of Multi-DOF Angle Measurement

**Figure 5:**
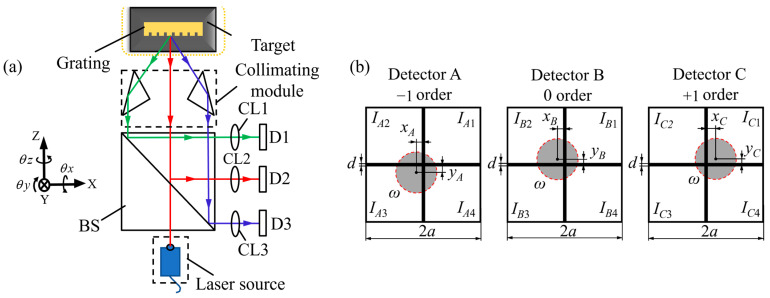
Schematic diagram of the basic principle of 3-DOF angular measurement grating encoders: (**a**) Optical configuration of a multi-axis angular grating encoder; (**b**) Diffraction spot distributions on the detectors.

Multi-DOF angular grating encoders build upon the principle of autocollimation by substituting the reflective mirror with a planar diffraction grating [253,254]. When illuminated by a collimated laser beam, the grating produces multiple diffraction orders whose directions are highly sensitive to changes in orientation [255]. This allows the system to detect pitch, yaw, and roll angles simultaneously [256]. The 0th-order beam, which follows the behavior of a specular reflection, is used to detect pitch and yaw based on its lateral displacement on a position-sensitive detector as the grating tilts about the X or Y axis. The ±1st-order beams, positioned symmetrically around the optical axis, respond to rotations around the Z axis. Their relative shift or rotation can be captured by quadrant photodiodes or imaging sensors to determine the roll angle. Figure 5 illustrates this measurement principle and the typical beam distribution used in multi-DOF angular detection based on diffraction.

These encoders achieve high-resolution angle measurement by exploiting the diffraction behavior of a two-dimensional grating illuminated by a collimated laser. When the beam strikes the grating, it generates 0th and ±1st-order beams, which are directed onto three separate detectors labeled A, B, and C, as illustrated in Figure 5b. The 0th-order beam is projected onto detector B, while the +1st and −1st-order beams are projected onto detectors A and C, respectively. The angular orientation of the grating directly affects the spatial positions of the diffracted spots on the three detectors. Pitch and yaw angles cause the 0th order beam to deviate laterally, resulting in a displacement of the spot on detector B. In contrast, roll angle induces a relative rotation between the +1st and −1st order beams, manifesting as opposite-direction displacements of the spots on detectors A and C. By precisely calculating the centroid positions $\left(x_{A},y_{A}\right)$, $\left(x_{B},y_{B}\right)$ and $\left(x_{C},y_{C}\right)$ of the spots on each detector, the corresponding angular variations around the X-, Y-, and Z-axes can be resolved in real time. The angular values are typically computed using a linear geometric transformation between spot displacements and angular rotations, expressed as:

(14) $$\theta_{x}=k_{x}\times y_{B}$$

(15) $$\theta_{y}=k_{y}\times x_{B}$$

(16) $$\theta_{z}=k_{z}\times \frac{x_{A}-x_{C}}{2}$$

where *k_x_*, *k_y_* and *k_z_* are angular sensitivity coefficients that depend on the system configuration, such as the focal length of the collimating optics, grating period, and propagation geometry. The pitch and yaw angles are inferred from the lateral shifts in the 0th order spot on detector A, while the roll angle is derived from the differential lateral displacement between detectors B and C. This formulation enables high-resolution and multi-axis angular measurement based on a single grating and compact optical system.

In practical multi-DOF angular grating encoder systems, accurate detection of diffracted spot positions is essential for resolving angular displacements around the three rotational axes [257]. Depending on system requirements, different types of photodetectors are used, each with specific strengths and limitations. Position-sensitive detectors (PSDs) offer high response speed and system simplicity by providing continuous analog signals for spot centroid estimation. They are well suited for dynamic tracking but have limited spatial resolution and are sensitive to beam shape variations, which can affect precision. Charge-coupled devices (CCDs) enable high-resolution centroid detection through digital image processing, making them ideal for applications requiring long-term stability and angular accuracy. However, their slower frame rates limit use in high-speed systems. Quadrant photodiodes (QPDs) offer fast response and high sensitivity by producing differential signals from four segments, which enables precise measurement of small angular changes. Their main limitation is the small active area, which restricts the measurable range. QPDs are thus best suited for high-resolution, narrow-range applications such as optical alignment and fine angular control. Therefore, selecting an appropriate photodetector should be determined based on the specific demands of the target application scenario.

### 3.2. Representative Architectures of Multi-DOF Angle Grating Encoders

#### 3.2.1. PSD/CCD Based Multi-DOF Angle Grating Encoders

**Figure 6:**
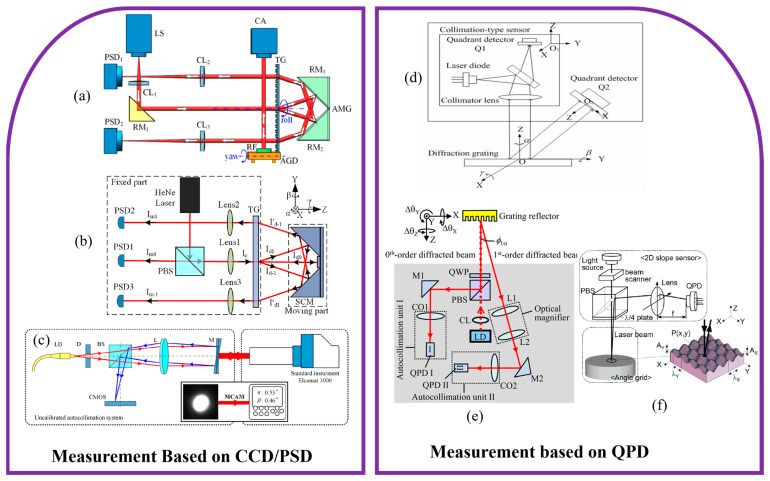
Representative measurement architectures for 3-DOF angle grating encoders: (**a**) Transmission grating-based differential autocollimator for roll sensor [258]; (**b**) Three-axis transmission-grating autocollimator [259]; (**c**) CNN-enhanced autocollimation angular sensor [260]; (**d**) Diffraction-grating encoder for angular and straightness sensing [261]; (**e**) Three-axis diffraction-grating angular encoder based on autocollimation [262]; (**f**) Integrated multi-axis grating encoder with sub-micro-arcsec angular resolution [263].

Position-sensitive detectors and charge-coupled devices have become widely adopted in multi-DOF angular grating encoders due to their high integration potential, flexible optical layouts, and relatively large detection ranges. Compared with quadrant photodiodes, which offer excellent sensitivity but are limited to narrow angular ranges, PSD and CCD based systems are more suitable for applications requiring wide-angle motion tracking. PSDs provide continuous analog output with rapid response, making them advantageous for dynamic measurements. CCDs, in contrast, offer high-resolution imaging capabilities that enable accurate position extraction of multiple diffracted spots through image processing algorithms. Both types of detectors can detect the lateral displacements of 0th- and ±1st-order beams generated from the grating surface, supporting the simultaneous reconstruction of pitch, yaw, and roll angles. Figure 6 summarizes the typical architectures employed for multi-axis angular measurement using these detector types.

As shown in Figure 6a, in 2019, Ren, W. proposed a resolution-enhanced roll-angle measurement system based on a transmission grating autocollimator. In this setup, the measurement beam passes through the rotating grating twice, resulting in doubled angular sensitivity [258]. The system achieved a resolution of 0.1 arcsec and stability of 0.11 arcsec, and effectively suppressed laser drift effects through differential detection. However, due to limitations in diffraction angle and grating size, the system was less suitable for long-stroke applications. As shown in Figure 6b, in 2022, the same research group introduced a three-dimensional laser autocollimator incorporating a transmission grating and a combined reflector [259], enabling simultaneous measurement of yaw, pitch, and roll angles by tracking three diffracted beams. Experimental validation demonstrated angular resolutions better than 0.01 arcsec, with repeatability values of 0.013 arcsec, 0.012 arcsec, and 0.009 arcsec for yaw, pitch, and roll, respectively. The system effectively reduced coupling errors among axes, making it well suited for ultra-precision alignment and calibration tasks.

To mitigate nonlinear errors arising from optical aberrations and misalignment, as shown in Figure 6c, in 2022, Shi, J. proposed an autocollimation method based on a multi-scale convolutional neural network [260]. By learning the complex relationship between beam spot features, such as shape and intensity profile and the corresponding angular deviation, the system achieved an expanded uncertainty of 0.29 arcsec with a coverage factor of k = 2, representing nearly a sevenfold improvement over conventional linear model. This learning-based method enhanced angular accuracy without increasing optical complexity and demonstrated strong generalization across different system configurations.

In 2024, Wu, Y. introduced a CMOS-based speckle imaging method for high-precision angular measurement over a large range [264]. By correlating the centroid positions of laser speckle patterns with angular displacements and calibrating the mapping relationship, the system achieved a measurement range of ±10,000 arcsec with error rates below 0.33%. This method outperformed conventional CCD and QPD based angular encoders in both measurement range and robustness. Its compatibility with neural network algorithms for speckle pattern recognition and error compensation further enhances its adaptability in challenging environments.

In summary, PSD and CCD based multi-axis angular grating encoders provide a practical and modular approach for measuring pitch, yaw, and roll angles over relatively wide dynamic ranges. By tracking multiple diffracted beams, these systems enable precise angular reconstruction and support integration with advanced signal processing methods. Recent studies have explored the use of neural network algorithms to improve measurement robustness, particularly in compensating for system nonlinearities, alignment errors, and environmental influences. However, image-based systems such as those using CCDs may introduce processing delays, which can limit their applicability in fast control loops. Additionally, high-resolution sensors and the associated computational load can increase system cost and design complexity. As such, while PSD and CCD based encoders offer excellent measurement capabilities, their use in engineering applications should be balanced with considerations of latency, stability, and system-level integration.

#### 3.2.2. QPD Based Multi-DOF Angle Grating Encoders

QPD based angular grating encoders are widely employed in precision angle measurement due to their compact form factor, high sensitivity, and fast response. By detecting the displacement of a diffracted light spot on a four-quadrant sensor, these systems can resolve angular variations with excellent resolution and minimal latency. The simple circuitry and low power consumption of QPDs make them particularly well suited for real-time, embedded applications where space is limited and high dynamic performance is required. Their ability to provide direct analog output also facilitates rapid signal processing in closed-loop control systems.

As shown in Figure 6d, in 2006, Liu, C. introduced a compact four-degree-of-freedom sensor combining a reflective diffraction grating with a QPD to simultaneously measure straightness and three angular errors [261]. The system utilized 0th- and +1st-order diffracted beams, with their spot displacements captured by a collimation sensor and a QPD, respectively. Although originally designed to integrate with a linear encoder for five-degree-of-freedom measurement, this configuration established a foundation for QPD-based angular sensing, demonstrating its potential in compact and integrated multi-axis encoder systems. Building on this concept, as shown in Figure 6e, Gao, W. in 2011 developed a three-axis autocollimator using a laser diode source (λ = 685 nm) and a grating reflector with a 1.7 μm pitch [262]. The reflected 0th-order beam was used for detecting pitch and yaw angles, while the ±1st-order beams, magnified through a 4.1× optical setup, were employed to detect roll. The system achieved a minimum detectable sinusoidal angular motion of 0.01 arcsec across all three axes, validating the high sensitivity of QPD-based autocollimation for angular metrology.

As shown in Figure 6f, in 2019, Shimizu, Y. described a suite of state-of-the-art multi-axis sensors, including three-axis autocollimators and 6-DOF planar encoders, based on planar diffraction gratings and QPDs [263]. The study emphasized the importance of combining ultra-high-resolution angular sensing better than 0.001 arcsec with advanced planar grating fabrication technologies. It also discussed approaches to expand measurement ranges, such as using mosaic gratings and multi-beam sensor heads. Furthermore, the author highlighted the increasing challenges of maintaining measurement accuracy as the number of degrees of freedom increases, particularly due to Abbe errors and alignment imperfections. QPD-based encoders, by directly tracking spot positions, partially mitigate these errors and improve angular sensing robustness. Thanks to their rapid analog response and seamless integration with diffractive optical paths, QPD-based angular encoders are often employed in conjunction with planar displacement sensors. When combined with three-axis grating displacement measurement modules, they enable full 6-DOF spatial metrology. The integration of translational and rotational sensing capabilities will be elaborated in subsequent sections.

Despite their advantages in resolution and system integration, QPD-based angular encoders face inherent limitations. The measurable angular range is constrained by the physical size of the sensor’s active area, restricting its use to small-angle applications. Moreover, QPDs are less capable of handling distorted or irregular beam profiles compared with imaging-based detectors, which can reduce measurement robustness under non-ideal optical conditions.

## 4. Grating Encoders for Combined Displacement and Angular Measurement

### 4.1. Multi-DOF Spatial Measurement Grating Encoders

As the demands of precision manufacturing and complex motion control continue to rise, the capability to accurately measure both spatial position and angular orientation has become a critical requirement. In applications such as semiconductor lithography, ultra-precision machining, and aerospace component alignment, motion platforms must operate within sub-micrometer tolerances while maintaining rotational stability. These conditions necessitate encoder systems that can resolve multiple degrees of freedom simultaneously, without sacrificing resolution, stability, or compactness.

**Figure 7:**
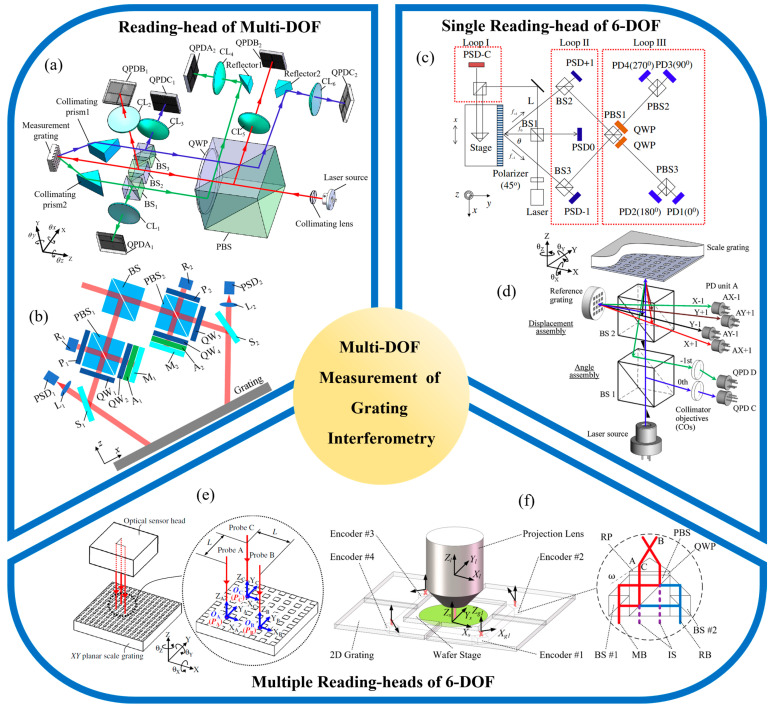
Representative measurement architectures for multi-DOF grating encoder: (**a**) Dual-module multi-DOF grating encoder [267]; (**b**) Integrated 5-DOF grating encoder [265]; (**c**) DOE-assisted multi-DOF precision grating encoder [269]; (**d**) Compact 6-DOF surface encoder for planar motion systems [270]; (**e**) Compact 6-DOF grating encoder with triple laser probes [271]; (**f**) High-speed 6-DOF grating encoder for wafer stage tracking [194].

Expanding on the foundational techniques for displacement and angular sensing discussed in previous sections, this part focuses on grating encoder designs that integrate both capabilities into a unified optical system. These multi-DOF encoders typically employ a combination of grating interferometry for displacement detection, autocollimation principles for angular tracking, and diffraction-based spot analysis to extract fine motion components. The resulting systems enable simultaneous monitoring of translational motion along the X, Y, and Z axes and angular variations such as pitch and yaw, all within a compact optical module. Their integrated architecture supports real-time pose reconstruction while minimizing space and alignment requirements, making them particularly suitable for embedded high-performance stages. Representative configurations of these integrated multi-DOF grating encoders are shown in Figure 7, which illustrates typical optical layouts that support simultaneous sensing of multiple spatial degrees of freedom.

As shown in Figure 7b, in 2020, Lv, Q. proposed a five-DOF grating-based precision metrology system that integrates two-dimensional displacement tracking and three-axis angular measurement within a symmetric Littrow configuration [265]. By employing a one-dimensional high-density grating and heterodyne interferometry, the system achieves large-range displacement sensing along both the grating vector and normal directions. Angular errors in pitch, yaw, and roll are extracted by analyzing variations in the ±1st-order diffracted beams using high-resolution position-sensitive detectors. The experimental results demonstrated displacement resolution better than 4 nm and angular resolution exceeding 1 arcsec. The decoupling between translational and angular DOFs, along with scalability enabled by grating size, makes the system well suited for high-precision industrial applications.

In 2022, Wang introduced an absolute-type four-DOF grating encoder capable of simultaneously measuring three angular components (*θx*, *θy*, *θz*) and vertical displacement (Z) [266]. The architecture employs a fixed reading head and a movable grating reflector, where incident light is diffracted into the 0th, +1st, and −1st orders and directed onto dedicated QPDs. Through spot position analysis and coordinate decoupling algorithms, the system realizes independent and absolute determination of all four DOFs. Experimental validation confirmed sub-μm resolution in the Z direction and sub-arcsec resolution in angular measurements. The implementation of a homogeneous error compensation matrix further suppressed installation-related crosstalk, enhancing system robustness. This design represents the first demonstration of true absolute 4-DOF pose sensing in a grating-based encoder, with significant potential for applications in synthetic aperture optics and precision assembly.

Further advancing this concept, as shown in Figure 7a, Luo in 2025 developed a high-precision grating encoder targeting out-of-plane multi-DOF posture sensing in in-plane displacement platforms [267]. The system introduces a defocused QPD model combined with a course–fine integration algorithm for enhanced beam spot localization across a wide angular range. A dual-module configuration incorporates a large-angle monitoring unit alongside a high-resolution narrow-range sensor, enabling angular resolution better than 0.04 arcsec and displacement resolution below 50 nm. Across a working range of ±10,000 arcsec in three angular axes and ±1.5 mm in Z, the system demonstrated excellent repeatability, low nonlinearity, and high measurement stability. These characteristics highlight the sensor’s suitability for advanced optical metrology and ultra-precision motion control over extended working ranges.

### 4.2. Six-DOF Spatial Measurement Grating Encoders

#### 4.2.1. Single Reading-Head 6-DOF Grating Encoders

Single reading-head 6-DOF grating encoders are designed to simultaneously measure three translational and three rotational motions using a single compact sensor head. By combining interferometric methods, diffraction optics, and multi-spot detection strategies, these systems can achieve full spatial pose measurement without requiring multiple detectors or mechanically complex assemblies. This approach is particularly suitable for space-constrained environments, high dynamic motion systems, and integration into precision positioning platforms.

In 2001, Bae, E. developed an early six degree of freedom measurement system based on beam deflection and triangulation [268]. A diffraction grating served as the reflective target, and the positions of the zeroth and first order diffracted beams were recorded using three two-dimensional position-sensitive detectors. A kinematic model was established to reconstruct the complete spatial pose of the target. The system demonstrated a translational resolution of 0.2 μm and an angular resolution of 0.5 arcsec. The maximum measurement errors were within ±0.5 μm and angular crosstalk was within ±2 arcsec. This work provided a foundational verification of the feasibility of achieving full 6-DOF sensing using a single sensor unit. Building on this foundation, as shown in Figure 7c, in 2011, Lee, C. presented a more advanced encoder system with improved precision [269]. This design included a diffractive optical element, a corner cube reflector, four position sensitive detectors, and a circularly polarized interferometric structure. The system could simultaneously resolve displacement and angular motion, providing better than 0.4 nm resolution for X-axis translation and angular resolution finer than 0.03 arcsec. The encoder was implemented on both piezoelectric and ball screw driven stages, confirming its adaptability to diverse motion systems and its effectiveness in capturing minor motion errors in ultra-precision environments.

A subsequent study in 2012 further examined the uncertainty of the system [272]. It identified key error sources including environmental instability, alignment deviations, and imperfections in optical components. Under controlled laboratory conditions, the encoder-maintained resolution at the sub-nanometer and sub-arcsec level. The dominant contributor to uncertainty was found to be environmental variation, particularly in the translational direction. These findings highlighted the necessity of system-level calibration and environmental compensation for long-term measurement reliability.

As shown in Figure 7d, in 2013, Li, X. introduced a surface encoder that combined a planar scale grating with a compact optical sensor head [270], using a shared laser source with a wavelength of 405 nm. The system simultaneously measured displacement in three axes through grating interferometry and detected angular motion by tracking diffracted beam positions. The sensor head measured 95 mm by 90 mm by 25 mm, and the grating had a pitch of 0.57 μm. The experimental results showed a translational resolution of 2 nm and angular resolution between 0.1 and 0.3 arcsec. The encoder design was optimized for compatibility with planar stages and demonstrated low interpolation errors and minimal crosstalk, offering advantages for compact precision systems. In 2015, Hsieh, H. proposed a unified optical structure that integrated heterodyne interferometry, grating shearing, and Michelson interferometry [218]. The system utilized three identical sensor units to measure translational displacement along X, Y, and Z axes, and derived angular values by comparing phase shifts between detection paths. The system achieved displacement resolution of 2 nm and angular resolution of approximately 0.01 arcsec. This architecture demonstrated the potential of combining multiple interferometric methods to enhance measurement robustness and scalability in a single sensor platform. Then, in 2021, Yu, K. developed a dual-probe measurement architecture using a common optical system to simultaneously monitor the 6-DOF motion of two closely spaced components. Each probe included a three-axis grating interferometry unit and a three-axis diffraction-based autocollimator. By sharing the optical path, the system reduced sensitivity to environmental noise. The experimental system demonstrated angular resolution of 0.02 arcsec and translational resolution in the range of 50 to 100 nm. Calibration, current-stabilized laser sources, and spectral compensation algorithms were employed to suppress error sources including grating imperfections and stray interference. The system achieved repeatability at the submicron and sub-arcsec level across multiple channels.

These developments highlight the ongoing progress in single reading-head six-degree-of-freedom grating encoders, with a clear focus on enhancing integration, measurement precision, and system reliability. Such encoders offer notable benefits, including a compact design, lower sensitivity to installation errors, and suitability for embedded applications. However, challenges still exist in maintaining performance under varying environmental conditions, ensuring long-term operational stability, and managing the effects of motion coupling between different axes.

#### 4.2.2. Multi Reading-Head 6-DOF Grating Encoders

While single reading-head grating encoders offer a compact approach to six-degree-of-freedom pose measurement, their use can be limited by geometric coupling, restricted range, and reduced flexibility in large-scale or high-speed systems. To overcome these challenges, multi reading-head architectures have been introduced. These systems use several sensor heads placed at different positions on a common grating surface, each measuring local displacement with high resolution. By combining data from all heads through rigid-body kinematic modeling, the full spatial pose, including position and orientation, can be accurately reconstructed. This method improves angular resolution due to increased spacing between heads, enhances system robustness, and supports more flexible mechanical designs. The grating target is typically planar or patterned, and each head employs techniques such as diffraction-based interferometry or optical spot tracking for displacement sensing.

In 2009, ASML implemented a laser grating interferometer in its NXT lithography platform, employing a cross-shaped arrangement of four dual-axis reading-heads [273]. Each sensor provided x and z displacement signals, yielding a total of eight independent channels for reconstructing full 6-DOF stage motion. Displacements along the z-axis were referenced to the laser wavelength, while x and y displacements were derived from the grating pitch of 1 µm. The system achieved a displacement resolution of 0.22 nm and angular resolutions of 0.001 arcsec in X and Y directions, and 0.0007 arcsec in the z direction, fully meeting the positioning requirements of 7 nm technology node lithography. As shown in Figure 7e, in 2014, Li, X. proposed a surface encoder utilizing planar grating interferometry with three parallel laser probes [271]. These probes were projected onto a measurement grating and a reference grating featuring identical microstructures. Each probe generated four interference signals, from which three-axis displacement (Δ*x*, Δ*y*, Δ*z*) was extracted. Angular errors (*θx*, *θy*, *θz*) were simultaneously determined from displacement differences and probe geometry. The prototype system demonstrated sub-nanometer translational resolution and angular accuracy better than 0.1 arcsec.

As shown in Figure 7f, in 2019, Zhu, Y. developed a fast 6-DOF computation algorithm based on phase-shifted grating interferometry for real-time motion tracking in wafer stages [194]. The algorithm incorporated affine transformation principles to model changes in diffraction spot positions and optical path lengths. A polynomial approximation was introduced to reduce computational complexity while preserving accuracy. Both The experimental results and ZEMAX simulations validated the model’s effectiveness, achieving translational errors below 10 pm and update times as fast as 1.7 µs. The same year, Zhu’s group addressed geometric coupling effects such as rotation-induced displacement error, Abbe offset, and cosine error through an offline calibration process [216]. By constructing an overdetermined system using redundant measurement channels and solving for geometric nonlinearities, they significantly enhanced system accuracy. The calibrated system achieved picometer-level displacement precision and was capable of real-time operation without sacrificing performance.

In 2024, Wen introduced an absolute-type 6-DOF evaluation method for precision rotary axes [274]. The technique employed multi-wavelength phase-shifting interferometry in conjunction with a specially fabricated micro/nanostructured artifact that was aligned with the rotational axis. This configuration enabled synchronized extraction of all six motion error components based on spatial pose variations. Experiments conducted on an Aerotech air-bearing rotary stage demonstrated angle positioning errors of 2.98 arcsec, tilt errors of 0.91 arcsec, a radial runout of 399 nm, and axial motion of 37 nm. The system supported dynamic and comprehensive motion error analysis, outperforming conventional methods limited to single-degree measurements.

These advancements collectively highlight the potential of multi reading-head grating encoder systems in delivering high-accuracy, real-time six-dimensional motion sensing. With appropriate geometric modeling, optical configuration, and calibration techniques, such systems have become indispensable tools in applications demanding sub-nanometer and sub-arcsec precision.

The progress of 6-DOF grating encoders demonstrates a clear balance between resolution, measurement range, system complexity, and practical implement ability. Single reading-head architectures provide excellent compactness and ease of integration, achieving sub-nanometer translational resolution and sub-arcsecond angular resolution within a unified optical module. Their simplicity makes them highly attractive for embedded stages and space-limited environments. However, they remain more vulnerable to cross-axis coupling, restricted range, and environmental disturbances, which may limit long-term stability in demanding applications. Multi reading-head configurations alleviate these issues by distributing probes across larger grating areas, thereby enhancing angular resolution, extending range, and improving robustness against noise and misalignment. At the same time, they introduce higher mechanical and optical complexity, require precise calibration and kinematic modeling, and increase integration cost. From an engineering perspective, single-head encoders are better suited for compact motion platforms and applications requiring minimal hardware footprint, whereas multi heading head designs are more appropriate for semiconductor lithography, large-scale optical instruments, and aerospace systems where long-range precision and stability are critical. These trade-offs indicate that the choice of architecture must be guided by application-specific priorities, balancing the need for accuracy, robustness, and ease of deployment.

### 4.3. Coupling Errors of Multi-DOF Measurement Grating Encoders

As grating encoders are extended to support multi-DOF spatial measurements, a key technical challenge is the mitigation of coupling errors between different motion axes [275]. Unlike traditional single-axis systems, where each degree of freedom is measured independently, multi-DOF encoders are more susceptible to inter-axis interference due to shared optical paths, overlapping detection regions, and non-ideal mechanical alignments errors. Translational movements can affect angular measurement outputs, and small rotations may introduce errors in displacement readings. These coupling effects are especially evident in integrated encoder configurations that rely on diffraction tracking, autocollimation, or optical spot detection to sense multi-DOF simultaneously. In practical systems, alignment errors, grating imperfections, and mechanical deformation further increase the risk of cross-talk between channels.

In 2019, Chang, D. investigated the formation of coupling errors in a spatially separated dual-diffraction heterodyne grating interferometer, which was developed to address frequency aliasing caused by imperfect polarization beam splitting [276]. By employing spatially isolated optical paths, the system effectively suppressed unintended frequency mixing and improved resistance to external optical interference. Building on this concept, Wang, G. in 2024 introduced a quasi-common-path heterodyne interferometric system featuring oblique beam incidence to reduce periodic nonlinearity [246]. Their design achieved a residual error of just 0.3 nm, demonstrating strong environmental robustness and suitability for demanding applications such as scanning beam lithography and ultra-precision stage motion control.

Gao, W. investigated the cross-axis interference in a 6-DOF surface encoder that employed a planar scale grating in combination with a reference grating. Translational displacements were derived from diffracted beam overlap between the two gratings, while angular motions were extracted solely from the scale grating. However, due to imperfections in polarization components, stray light from the reference grating leaked into the angular sensing path, producing substantial cross-talk. To resolve this issue, the angular detection module was repositioned and the cube beam splitter was replaced with a plate-type splitter. The experimental results demonstrated a reduction in angular cross-talk from 3 arcsec to 0.02 arcsec, highlighting the importance of optical layout optimization in suppressing inter-axis interference and improving overall measurement accuracy.

As multi-DOF grating encoders continue to advance in integration and functionality, resolving coupling errors through both optical system design and signal processing strategies remains imperative. Progress in component alignment accuracy, optical path stabilization, and real-time data synchronization will play a critical role in ensuring reliable six-axis pose sensing for next-generation manufacturing, metrology, and scientific instrumentation.

## 5. Conclusions and Prospect

This review provides a detailed and structured summary of grating encoder technologies for multi-DOF spatial measurement, covering both translational and angular sensing. By categorizing system architectures into normal-incidence, Littrow-incidence, absolute-type planar encoders, and three-dimensional configurations involving prisms, diffractive elements, and lens-based or integrated modules, it outlines the diverse pathways through which grating interferometry has been applied. The field has progressed steadily from basic two-axis systems toward full six-degree-of-freedom encoders capable of sub-nanometer resolution in displacement and sub-arcsec precision in angle measurement. Both single-head and multi-head configurations have demonstrated accurate pose determination, supported by advances in optical layout and signal-processing techniques.

Despite these developments, several technical challenges remain. These include coupling between motion axes, phase nonlinearity, sensitivity to environmental factors such as temperature and vibration, and limitations in achieving long-range absolute measurements. Additionally, the complexity of optical alignment and system calibration tends to increase with the number of measured degrees of freedom, posing challenges to system integration and modular design. Addressing these issues will require further improvements in component quality, enhanced signal demodulation algorithms, and robust error compensation strategies. Continued research in these areas is essential to support the deployment of grating encoders in demanding industrial and scientific applications.

Future progress in multi-DOF grating encoders is expected to follow several key directions. Miniaturization of optical modules, the adoption of intelligent signal processing, and the integration of hybrid sensing technologies will be central drivers of innovation [277,278,279]. Advances in diffractive optics and nanofabrication will enable the development of planar gratings, beam splitters, and compact optical elements with high thermal and mechanical stability [280]. These features are particularly advantageous in space-constrained applications such as robotic arms, precision manufacturing stages, and aerospace systems [280]. In parallel, intelligent algorithms based on machine learning are expected to improve signal decoding and error correction [281]. Deep learning methods can capture nonlinear relationships between measurement axes and effectively mitigate environmental noise and system imperfections, paving the way for adaptive absolute encoding schemes that optimize spatial resolution, robustness, and decoding accuracy [282,283,284]. Several emerging technologies are likely to reshape the field. Metasurface-based gratings provide unprecedented control over diffraction phase and amplitude, opening opportunities for ultra-thin, multifunctional encoder components with tailored optical responses [285,286]. Integrated photonic encoders, which leverage on-chip light sources and detectors, promise scalable, low-cost platforms with improved stability and alignment tolerance [287]. Meanwhile, advances in quantum-enhanced metrology suggest that entangled or squeezed light sources could further push the limits of resolution and noise suppression, particularly in environments where conventional optical methods are constrained. The combination of these technologies with established grating-based approaches offers a compelling route toward compact, high-performance encoders with capabilities beyond the current state of the art [288].

In addition to these technological advances, the industrial application outlook highlights the pivotal role of multi-DOF grating encoders across diverse engineering domains. In semiconductor manufacturing, the demand for sub-nanometer stage positioning in lithography and wafer inspection makes grating encoders particularly attractive due to their high resolution and thermal stability, offering a scalable alternative where interferometers face integration and cost limitations [289]. In precision machine tools, their capability to deliver real-time multi-axis feedback supports active error compensation and adaptive machining, thereby enhancing throughput and accuracy under industrial conditions [216]. For large optical instruments such as astronomical telescopes and interferometric observatories [290], grating encoders enable precise multi-axis alignment of mirrors and structures over wide ranges while maintaining reliability in the presence of thermal fluctuations and mechanical deformation [291]. Across these scenarios, their combination of compact form, high accuracy, and integrability addresses key challenges in stability, calibration, and system interoperability, reinforcing their importance as an enabling technology for next-generation industrial and scientific platforms [292].

At the same time, hybrid metrology systems that combine interferometric sensing with image-based techniques such as autocollimation, speckle tracking, and structured light are expected to further improve measurement resilience and versatility [293]. These systems can simultaneously capture displacement and angular variations while reducing sensitivity to environmental disturbances [294]. As encoder applications expand to larger ranges and more complex motion paths [295], advances in real-time synchronization [296], parallel data processing [297], and modular hardware design will be essential to ensure accuracy and system robustness [297,298]. Taken together, these developments suggest that grating-based encoder technologies are poised to become a cornerstone of future precision measurement, capable of providing high-resolution, real-time, and multi-dimensional motion feedback in compact and scalable architectures. Realizing this potential will require close collaboration among specialists in optics, mechanics, electronics, and computational methods to drive continued innovation [110].

## Figures and Tables

**Table 1:** Comparison of grating interferometer architectures for 2D displacement sensing.

Parameters	Normal-Incidence Configuration	Littrow-Incidence Configuration
Incident Angle	90°	Equal and opposite Littrow angles
Beam Path Geometry	Incident and diffracted beams propagate along overlapping or intersecting paths within a symmetric layout	Incident and diffracted beams follow folded, counter-propagating paths that are spatially separated
Sensitivity to Out-of-Plane Motion	Sensitive to out-of-plane motion and typically requires calibration for compensation	Inherently resistant to Z-direction displacement with minimal sensitivity
System Integration Complexity	Relatively simple alignment, but susceptible to drift	Requires precise angle alignment and high-quality reflective optics
Typical Resolution	~1–5 nm	<1 nm
Angular Tolerance	Angular tolerance is limited, particularly sensitive to misalignment in multi-axis setups	Provides higher angular tolerance and maintains accuracy under slight misalignments

**Table 2:** Comparison of four collimation strategies in grating encoders.

Collimation Strategy	Z-Axis Sensitivity (μm)	Working Distance (mm)	Integration & Compactness
Prism Group Collimation	50–500	5–20	Moderate
Diffraction Device Collimation	-	10–50	Compact
Lens Group Collimation	<10	>80 mm achievable	Easy to adjust
Optical System Collimation	50–200	10–20	Compact

## Data Availability

The data presented in this study are available on request from the corresponding author.
